# Comparative analysis of stress distribution in residual roots with different canal morphologies: evaluating CAD/CAM glass fiber and other post-core materials

**DOI:** 10.1186/s12903-024-04109-9

**Published:** 2024-03-15

**Authors:** Minghao Huang, Biyao Wang, Kaige Zhang, Xu Yan, Zhiyu Chen, Xinwen Zhang

**Affiliations:** 1 Department of Oral Implantology, School and Hospital of Stomatology, Liaoning Provincial Key Laboratory of Oral Diseases, China Medical University, No.117 North Street Nanjing Road, Shenyang, 110002 China; 2 The VIP Department, School and Hospital of Stomatology, Liaoning Provincial Key Laboratory of Oral Diseases, China Medical University, Shenyang, China; 3 Department of Prosthodontics, Hebei Key Laboratory of Stomatology, Hebei Clinical Research Center for Oral Diseases, School and Hospital of Stomatology, Hebei Medical University, Shijiazhuang, China; 4 Laboratory Animal Centre, School and Hospital of Stomatology, China Medical University, Shenyang, Liaoning China

**Keywords:** CAD/CAM, Dental materials, Finite element analysis, Fiber post, Post-and-core, Root canal

## Abstract

**Background:**

The selection of post-core material holds significant importance in endodontically treated teeth, influencing stress distribution in the dental structure after restoration. The use of computer-aided design/computer-aided manufacturing (CAD/CAM) glass fiber post-core possesses a better adaptation for different root canal morphologies, but whether this results in a more favorable stress distribution has not been clearly established.

**Materials and methods:**

This study employed finite element analysis to establish three models of post-core crown restoration with normal, oversized, and dumbbell-shaped root canals. The three models were restored using three different materials: CAD/CAM glass fiber post-core (CGF), prefabricated glass fiber post and resin core (PGF), and cobalt-chromium integrated metal post-core (Co-Cr), followed by zirconia crown restoration. A static load was applied and the maximum equivalent von Mises stress, maximum principal stress, stress distribution plots, and the peak of maximum displacement were calculated for dentin, post-core, crown, and the cement acting as the interface between the post-core and the dentin.

**Results:**

In dentin of three different root canal morphology, it was observed that PGF exhibited the lowest von Mises stresses, while Co-Cr exhibited the highest ones under a static load. CGF showed similar stress distribution to that of Co-Cr, but the stresses were more homogeneous and concentrated apically. In oversized and dumbbell-shaped root canal remnants, the equivalent von Mises stress in the cement layer using CGF was significantly lower than that of PGF.

**Conclusions:**

In oversized root canals and dumbbell-shaped root canals, CGF has shown good performance for restoration of endodontically treated teeth.

**Clinical relevance:**

This study provides a theoretical basis for clinicians to select post-core materials for residual roots with different root canal morphologies and should help to reduce the occurrence of complications such as root fracture and post-core debonding.

## Introduction

For endodontically treated teeth, posts confer restorations with stability and higher retention [1]. Posts come in various shapes and sizes, and they can be fabricated from metal alloys, glass fiber, or zirconia. Although metal alloy posts have high mechanical strength and excellent biocompatibility, the use of metal alloys is declining as such posts are not esthetically pleasing and they can lead to color changes in the remnants of the tooth due to corrosion [1]. In addition to better esthetic properties, the fatigue survival of fiber posts is similar [2] to that of metal alloy posts, while their modulus of elasticity is similar to that of dentin, thus providing a more favorable stress distribution and reducing the occurrence of fractures [3].

Nowadays, with patients having high esthetic demands, metal-free post-core systems that do not affect the color of the teeth have become one of the preferred options for restoring pulpless teeth [3]. In this approach, prefabricated glass fibers are becoming increasingly popular because they achieve satisfactory results while saving time and reducing costs [4]. In addition to the material, which determines the particular elastic modulus of the post, the diameter and height of the post contribute significantly to the fracture resistance of the restored tooth [5], which is closely related to the morphology of the root canal. In some funnel-shaped root canals, however, the limitations of preformed fiber posts have been exposed, as they cannot follow the shape of the root cavity in the most appropriate way in contrast to cast metal posts. As a result, the adhesive interface becomes a weak link between dentin and the fiber post, resulting in a higher failure rate of restorations [6]. The use of prefabricated fiberglass posts with composite resin to meet the anatomical morphology of the root canal is one way to overcome this problem [7]; however, this strategy multiplies the number of adhesive interfaces between the post and the root canal.

Against this background, attempts have been made to use computer-aided design/computer-aided manufacturing (CAD/CAM) glass fiber post-core, a fiber post material with ability to adapt to the morphology of the root canal. The earliest application to date is a case reported by Liu et al. [6] involving the restoration of a fractured anterior tooth, for which satisfactory results were obtained. Thereafter, the mechanical properties and superficial characterization of CAD/CAM glass fiber post-core (CGP began to be reported [8], and many researchers conducted in vitro mechanical experiments to explore the fracture resistance and failure modes of the material [9, 10]. With these studies as a foundation, clinical case reports of the application of CGP have increasingly been published [11, 12], and this material is becoming one of the optimal choices for the restoration of anterior fractures with large dental defects. In addition to the application of CGP in anterior teeth, clinical cases of CGP in premolars have been constantly reported [13, 14], and the more complex root canal morphology of premolars compared to anterior teeth seems to be more suitable for the application of CGP as well. However, the studies performed to date have mainly been concerned with the restoration of normal root canals in anterior teeth with this material, while attention has also been focused on the amount of remaining dentin tissue and the integrity of the ferrule. However, the morphology of the root canal is one of the most important factors in the selection of the post-core material for practical clinical applications [15], but few studies have been performed on the different morphologies of root canals restored with CGP.

As such, in this study, three-dimensional finite element analysis (FEA) was applied to explore stresses distribution in each component of restorations with CGP in residual roots with different morphological root canals. The aim of this work was to assess the stress promoted by residual roots with root canals of different morphologies (normal root canal, oversized root canal, dumbbell-shaped root canal) restored with CGP, prefabricated glass fiber post and resin core (PGF), or cobalt-chromium integrated metal post-core (Co-Cr) and a zirconia crown. The hypothesis tested here that different post-core systems will present different stress intensity and distribution patterns. In addition to FEA, a clinical case was reported to provide experience of the application of CGP for clinicians. This report describes the esthetic restoration of anterior teeth with a massive defect of an oversized root canal. CGP and zirconia crowns were applied for post-core crown restoration to achieve satisfactory esthetic results.

## Materials and methods

One standard maxillary central incisor extracted due to periodontitis, and one maxillary second premolar and one mandibular second premolar extracted due to the need for orthodontics were selected for Cone Beam Computed Tomography (CBCT) scanning (SkyScan 1174v2; Bruker Microct, Billerica, MA, USA) to obtain digital images in the DICOM format. The parameters of CBCT was performed with a voltage of 69 kV, an X-ray beam current of about 100 mA, and a resolution of 20 μm. The image data were imported into the reverse-engineering software Mimics Medical 17.0 (Materialise Medical, Leuven, Belgium) and Geomagic Wrap 2017 (Geomagic Inc., Utah, USA) to reconstruct the models of the three teeth.

The obtained models in standard for the exchange of product model data (STEP) format were imported into the CAD software Solidworks 2018 (Waltham, MA, USA) in order to construct the model of normal root canal, oversized root canal, and dumbbell-shaped root canal for post-core crown restorations, in accordance with the clinical specifications. The cross-sections of the posts in the normal root canal and dumbbell-shaped root canal groups were oval in shape, while the root canal taper and root canal opening were larger in the oversized root canal group. The apices of all models retained 4 mm of the remaining gutta-percha with a 2 mm dentin ferrule in the cervical region. The cut edge of the crown was set at 2 mm, and the axial wall thickness was not less than 1 mm. To investigate the main results and reduce the amount of calculation, the cortical bone, cancellous bone, gutta-percha, and periodontal ligament (0.2 mm) of the model were appropriately simplified.

Each model was further divided into three subgroups according to the post-core material and was repaired using Co-Cr, CGF, and PGF. It is worth noting that due to the dumbbell-shaped root canal morphology, the number of preformed fiber post in this group of models was 2. Since the PGF and the root canal do not fit together perfectly, the space between the PGF and the root canal is filled with cement. Except for Co-Cr where glass ionomer cement was chosen, resin cement was used for all of the cases. The thickness of the adhesive was 0.05 mm for bonding zirconia crowns and 0.1 mm for bonding post-cores.

All models were imported into the FEA software ANSYS Workbench 2021 R1 (Swanson Analysis, Canonsburg, PA, USA) for meshing and biomechanical analysis. The meshing of all models is obtained by the mesh sensitivity analysis method, and the orthogonal quality ranges from 0.8512 to 0.8573. After meshing, the number of elements was 1,405,312–1,411,849 and the number of nodes was 1,995,631–2,009,268. All materials were set to homogeneous isotropic linear elastic materials, except for the PGF, which was set to orthogonal anisotropy. The modulus of elasticity and Poisson’s ratio data were obtained from high-quality literature and imported into the software (Table 1). Bonding contacts were specified along with all model interfaces in order to simulate the interfaces between model components. The surface of the cortical bone was assumed to be rigidly fixed in the x, y, and z directions.

**Table 1:** Physical properties of the materials used

Material	Elastic modulus (GPa)	Poisson’s coefficient
Cortical bone [18]	13.7	0.3
Spongy bone [18]	1.37	0.3
Dentin [18]	18.6	0.31
Periodontal ligament [19]	0.0689	0.45
Gutta-percha [20]	0.00069	0.45
Prefabricated glass fiber post [20]	X = Y = 13.5 Z = 39	XY = 0.35 XZ = YZ = 0.285
Cobalt chromium [1]	211	0.42
CAD/CAM glass fiber post-core [1]	35	0.32
Resin core [19]	15.8	0.24
Glass ionomer cement [20]	4	0.35
Resin Cement [19]	11.54	0.24
Zirconia crown [19]	210	0.3

**Fig. 1:**
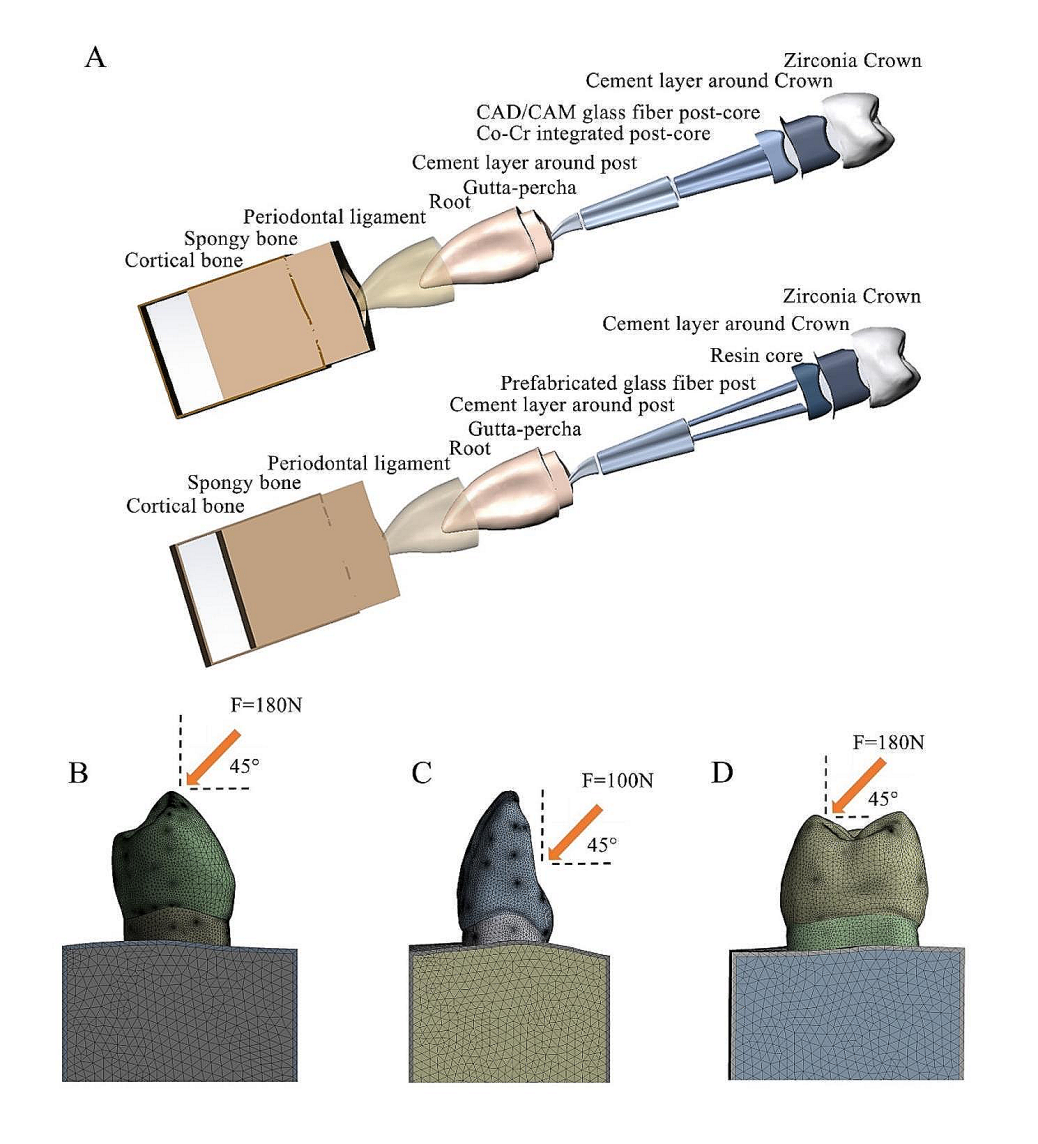
(A) Finite element analysis (FEA) components for dumbbell-shaped group repairing with CAD/CAM glass fiber/cobalt-chromium integrated metal post-core and prefabricated glass fiber post and resin core. (B) Visual model of Normal root canal group after static loading and meshing in FEA model. (C) Visual model of oversized root canal group after static loading and meshing in FEA model.D. Visual model of dumbbell-shaped root canal group after static loading and meshing in FEA model

A static occlusal force of 180 N at an angle of 45° to the longitudinal axis of the tooth was applied to the buccal cusps of the crowns of normal root canal [1]. In the case of dumbbell-shaped root canal groups, a 180 N force was applied to the palatal cusp surface of the crown, positioned 2 mm below the cusp tip, and at a 45° angle relative to the longitudinal axis of the tooth [16]. For the oversized root canal group, the loading point was located 2 mm from the incisal end of the crown and a static occlusal force of 100 N was applied, with the loading direction at an angle of 45° to the longitudinal axis of the tooth (Fig. 1) [17].

In this study, von Mises energy criteria were applied to evaluate the effects of loads on teeth and restorative structures using equivalent von Mises force and maximum principal stress. For each group of dental model components, the results of the stress distribution are presented as contour graphs with a color scale representing pressure in megapascals (MPa), and the stress concentrations in each dental model component are expressed in terms of the maximum equivalent von Mises stress (EVM) and the maximum principal stress (MPS). In addition, the color scale is used to enable comparisons between the analyzed models.

## Result

The EVM and MPS for roots, post-core, crown, cement layer around the post, and cement layer around the crown for each group are presented in Tables 2 and 3. Whether in the normal root canal group, oversized root canal group, or dumbbell-shaped root canal group, it was found that EVM and MPS of the post-core reached their maximum for Co-Cr and their minimum for PGF. The largest differences were found for the post with peak EVM of 185.99 MPa for Co-Cr, 34.23 MPa for CGF, and only 23.80 MPa for PGF in the normal root canal group. However, the different post-core materials did not result in evident differences in dentin of residual roots. Regardless of the morphologies of residual roots, the lowest EVM values were found in dentin of models using Co-Cr as the post-core material. For the cements for bonding posts, both EVM and MPS, which reflect the adhesive forces between the groups, showed the minimum values for Co-Cr and the maximum ones for PGF, with marked differences. The bonding cement applied to the PGF in the normal root canal group and the oversized root canal group was subjected to approximately 4.5 times the EVM compared with the cement applied to the Co-Cr. The cement of CGF was also subjected to greater stress than that of Co-Cr; however, compared with the value for PGF, EVM was reduced by 30%. This trend was even more pronounced in the dumbbell-shaped root canal group, in which EVM and MPS in the cement of PGF were 13.2 and 3.8 times higher than in the cement of CGF and Co-Cr, respectively. However, for the cement layers around crowns, there were no significant differences among the different groups and subgroups, for either EVM or MPS.

**Table 2:** Peak equivalent von Mises stress in normal root canal group, oversized root canal group and dumbbell-shaped root canal group within restored tooth components: root, post-core, crown, cement layer p, and cement layer c (MPa)

	Normal Root Canal Group			Oversized Root Canal Group			Dumbbell-shaped Root Canal Group		
	CGF	PGF	Co-Cr	CGF	PGF	Co-Cr	CGF	PGF	Co-Cr
Root	206.36	207.55	202.44	72.774	72.727	72.618	124.42	126.64	114.97
Post-core	34.288	23.802	185.99	14.776	12.605	45.022	25.276	14.271	124.77
Crown	386.7	389.85	357.4	60.783	69.773	54.53	163.72	161.47	169.61
Cement layer p	11.649	16.976	3.8416	2.9937	4.3468	0.94451	8.7653	33.329	2.5097
Cement layer c	4.3344	4.446	3.9895	2.1336	2.7365	0.80772	3.897	4.4172	2.4642

**Table 3:** Peak maximum principal stress in normal root canal group, oversized root canal group and dumbbell-shaped root canal group within restored tooth components: root, post-core, crown, cement layer p, and cement layer c (MPa)

	Normal Root Canal Group			Oversized Root Canal Group			Dumbbell-shaped Root Canal Group		
	CGF	PGF	Co-Cr	CGF	PGF	Co-Cr	CGF	PGF	Co-Cr
Root	305.49	307.5	299.3	90.162	90.284	89.6	116.38	117.86	109.51
Post-core	19.695	21.141	142.99	5.9645	7.2069	27.875	15.64	12.066	92.696
Crown	113.41	113.91	105.98	35.018	49.288	27.892	124.26	127.66	123.97
Cement layer p	6.1084	13.685	2.7704	1.0599	1.6532	0.56632	4.5313	23.4	1.707
Cement layer c	2.8624	2.7557	2.9279	2.281	2.9263	0.86601	3.7327	4.295	2.2453

**Fig. 2:**
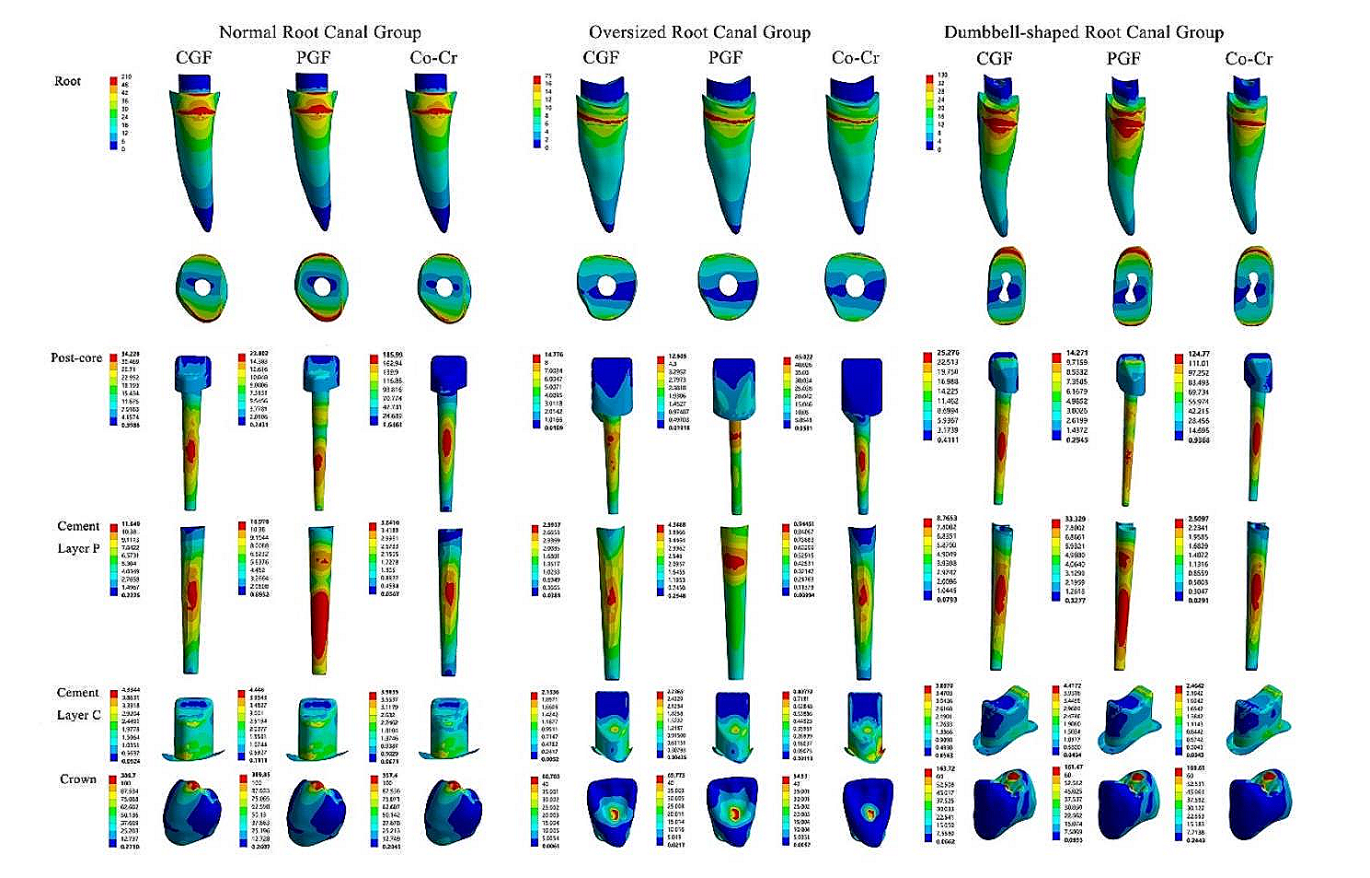
Distribution of von Mises stress in normal root canal group, oversized root canal group and dumbbell-shaped root canal group within restored tooth components: root, post-core, crown, cement layer c, cement layer CGF: CAD/CAM glass fiber post-core; PGF: prefabricated glass fiber post and resin core; Co-Cr: cobalt-chromium integrated metal post-core

Figure 2 shows the stress distribution of each component in the different groups. For the residual roots of the normal root canal group and oversized root canal group, there was little difference between the EVM and PM with the application of the three materials; both with stress in the order of Co-Cr < CGF < PGF and with similar stress distributions, the stresses were concentrated at the cervical-alveolar bone contact interface on the labial and lingual sides of the tooth, with the lingual side being larger than the labial side. However, in the dumbbell-shaped root canals, there was a marked difference in the area of the stress concentration zone in the residual roots, with the value for Co-Cr being significantly smaller than those for the remaining two groups. For the posts, the Co-Cr exhibited significant areas of stress concentration in all three groups, being located basically in the middle third of the post. The stress distribution of CGF in each group was similar to that of Co-Cr, but the stress was concentrated at a lower location, approximately in the third of the post where the root was located, while the stress distribution was more uniform. With the application of PGF repair, the stresses were concentrated in the neck third of the post in the oversized root canal group and in apical third of the post in the remaining two groups. The general trend of stress distribution in the cement layer of the post was similar to that in the post itself, with the stress distribution of applied PGF being particularly notable. In both oversized root canal and dumbbell-shaped root canal, the area with high stress was larger than that when the other two post-core materials were applied, and there were two stress concentration areas in both the neck third and the root third. As for the stress distribution in the crown bonding cement, the distribution within each group was similar for both EVM and MPS, independent of the choice of post-core material.

**Fig. 3:**
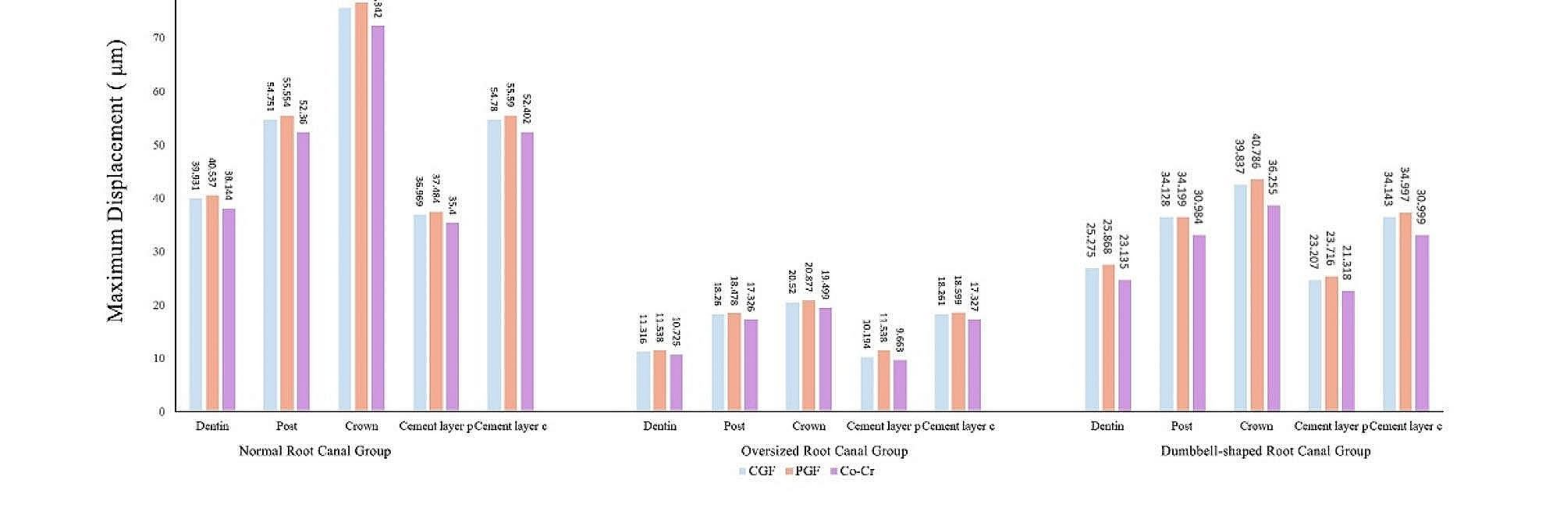
Maximum displacement in normal root canal group, oversized root canal group and dumbbell-shaped root canal group within restored tooth components: root, post-core, crown, cement layer c, cement layer p. CGF: CAD/CAM glass fiber post-core; PGF: prefabricated glass fiber post and resin core; Co-Cr: cobalt-chromium integrated metal post-core

Figure 3 shows the maximum displacement of the different components in each group. In the different groups, the cement layer showed the highest maximum displacement when using preformed glass fiber post for restoration: 37.4 μm in the normal root canal group,11.5 μm in the oversized canal group, and 23.7 μm in the dumbbell-shaped root canal group

## Discussion

Based on the results obtained, the tested null hypothesis was accepted in this experiment. This indicated that there are differences in the stress distribution of post-core restorations in residual roots with different root canal morphologies upon the application of three different post-core materials. With the use of the FEA method, we analyzed the force transfer and compared the stresses generated in the root, post-core, crown, and cement layer around the post and crown. This revealed the area with the maximum stress concentration as a reliable indicator of the area where restoration failure may begin.

Cast metal post-cores used to be the gold standard for post-core crown restorations and were routinely applied. However, in practice, they have been shown to be particularly susceptible to vertical root fractures [3], which eventually lead to tooth extraction. This can be attributed to the difference between the elastic modulus of the dentin and the elastic modulus of the post-core material. The modulus of elasticity is influenced by the chemical composition and structure of the material and is one of the material’s inherent properties. The results of several studies have shown that, when the material of the post has a high modulus of elasticity, the stresses in the dental tissue are not homogeneous and this tissue is subjected to destructive stresses [21]. Some researchers asserted that the ideal material for making a post is the one with an elastic modulus closest to that of dentin, which can range from 10 to 30 GPa depending on age and anatomy [22]. Therefore, glass fiber posts are widely used due to the reduced risk of root fracture [3]. It is expected that the associated disadvantage of metallic posts can be overcome by using a material with a lower modulus of elasticity to obtain a favorable root stress distribution while ensuring similar strength [23]. However, the results of this experiment showed that, for root dentin in each group, Co-Cr was resulted in similar EVM and MPS stress to those for CGF and lower than those for PGF. This may be related to the fact that all groups in this experiment involved models with intact dentin ferrules with minimum 2 mm height. As shown by Savychal et al., the presence of ferrules increases the stress on cervical dentin and accordingly decreases the bonding stress [24]. In this context, it remains to be confirmed whether residual roots without an intact ferrule can be restored using CGF.

In addition to the maximum stress, the uniformity of the stress distribution is an effective indicator of long-term clinical results in the application of post-core crown restorations [25]. In this study, the stress in CGF in the normal root canal group and dumbbell-shaped root canal group was concentrated downward compared with the findings for Co-Cr, being focused in approximately the apical third of the post. Compared with the other two post-core materials, CGF exhibited a uniform stress distribution without significant concentrations of stress. As numerous studies have shown, if stresses are concentrated in the coronal third of the root, there is an increased probability of post-core failure in that tooth [23, 26]. Therefore, as a type of fiber post, CGF possesses significant advantages in stress distribution compared with cast metal post-core. This is also consistent with the results of a fracture resistance test for CGF recently reported by Suzaki et al. [27]. Their study showed that teeth with CGF were more fracture-resistant and repairable even after fracture, so CGF was recommended for clinical use to protect vulnerable pulpless teeth.

Problems in post adhesion are is a common phenomenon due to the damage limit of the cement interface being surpassed due to the concentration of stress [25]. Verri et al. [28] and Li et al. [29] suggested that the fiber post forms a structure in which stress encompasses all of root-cement-post because its elastic modulus is close to that of dentin. When subjected to an external force, the stress will reach the post through the cement layer. Compared with PGF loading, the use of CGF in the current study was associated with the transmission of less stress to the dentin, with more of the stress being taken up by the post itself. The cemented interface in the post-core treatment is of great importance because it is the area that is prone to rupture under load. High stress concentrations can cause the loss of bonding of cemented interface. This can lead to mechanical failure, resulting in the loss of restoration and development of periapical lesions after bacterial invasion [30].

In terms of influencing retention, the diameter of the prefabricated post is second in importance only to the length [2], and it has been reported that some root canals have failed due to space enlargement, resulting in an irreparable restoration [31]. The use of preformed fiber posts must be accompanied by an increased amount of adhesive, both in oversized root canals and in dumbbell-shaped ones. As shown in previous studies, preformed fiber posts rely on their relatively low modulus of elasticity to allow a more uniform distribution of occlusal load across the root-dentin-cement interface and to transfer the location of maximum stress to the cervical bone level [32, 33]. Some studies have shown that, to reduce the stress intensity at the tooth repair interface, a repair material with an elastic modulus similar to that of teeth should be selected [34]. Though the use of prefabricated fiberglass posts with resin composite has addressed, to some extent, the problem of the suitability of post for root-flared canals [35]. However, a meta-analysis conducted by Silva et al. revealed that although both the use of customization with auxiliary posts and customization with resin composite outperformed noncustomized posts with a thick cement layer in flared canals, the former demonstrated superior results [36]. This may be due to the lack of adhesion between the resin composite and the post, which creates movement within the root canal and ultimately leads to failure. Therefore, instead of finding a more effective surface treatment and bonding solution for preformed fiber piles with resin liners, CGP still has an advantage.

Oversized root canals are common in teeth with trauma, intra-root resorption, decay left unrepaired for a long period, and some maxillary anterior teeth, while oval root canals are more common in premolars. At present, there is no clear definition of an oversized root canal, and how oversized root canal models are prepared varies from study to study [37, 38]. In this study, an oversized root canal was defined as a root canal with a width greater than half of the root canal diameter and a taper greater than 8.58° (15%). The adaptation of posts and post tract should be a key consideration when restoring these two types of affected teeth with post-core crowns. Since preformed fiber posts are prefabricated and limited in diameter, the space between the post and the dentin needs to be filled with resin or bonding cement. Roberts et al. [39] suggested that light-curing resins have a tendency to suffer from shrinkage deformation, which affects the suitability of the posts, and therefore are not recommended for bonding fiber posts. Although some researchers consider that mismatched post tract and post diameter as well as cement thickness do not affect the bond strength of the post [40, 41], most of them suggest that the post and post tract should be precisely matched, and that poor suitability will create a lever force between the post and the root canal, constituting one of the reasons why the roots are more prone to fracture [42, 43]. This is in line with our results, which showed 30% and 70% reductions in EVM and MPS of cement when applying CGF compared with the values for cement of PGF in oversized and dumbbell-shaped root canals, respectively. Likewise, in the normal form of root canals cement layer around post did not differ much in the maximum displacement between the CGF and PGF groups, whereas in the oversized root canal a discrepancy arose, with the PGF group being 13.2% of the maximum displacement of the CGF group. In previous research, the difference in maximum displacement was attributed to the modulus of elasticity possessed by the different post-core materials [44], whereas in this study, it was found that even if the post-core materials were the same, the difference in the thickness of the adhesive due to the different morphology of the root canals had an effect on the maximum displacement. It is interesting to note that in addition to the magnitude of the maximum displacement, Ryousuke et al. started to study the distribution of displacement vectors for the debonding model [44], which is a good direction of research because, it could be an indication of the development of vertical root fracture.

Valter et al. investigated the relationship between post adhesion and cement film thickness, luting cement, and post pretreatment, which indicated that the more cement between the post and dentin, the poorer the retention of the post [5]. First of all, air bubbles are likely to be created if the adhesive layer is too thick, and the bubbles or voids formed represent weaknesses in the material, making the post vulnerable to loosening [45]. In addition, high polymerization shrinkage and incomplete light curing due to the post being outside of the range of light are two other important factors for debonding [45, 46]. Therefore, CGF has a significant advantage over PGF in terms of bonding, resulting in a reduction in the failure rate of the restoration due to debonding of the post-core. The manufacturer of the CGF included in this study claims that it has a bending modulus of 25.0–45.0 GPa. However, compared with PGF, existing CGF lacks longitudinal fibers [18], so PGF was tested in vitro with damage loads of approximately 75.9 kg [47] and 534 N [48]. Considering the more complex secondary processing required for CGF, it may be a future direction for this material, as longitudinal fibers can transfer less stress to the repair material and provide high flexural and fatigue strength.

However, this experiment still has the following limitations. First, the selection of equivalent von Mises stresses and maximum principal stresses as the indexes of the study cannot fully reflect the state of reality of the stress distribution. Indeed the choice of metrics for FEA of brittle materials in endodontic restorations has long been debated [49]. Some authors use maximum principal stress to analyze the results, which is based on Rankine or maximum normal stress criteria for dentin failure [1, 20]. The majority of studies, including in this study, have selected the von Mises criterion, a simple failure criterion that can be directly comparable to tensile strength by using the equivalent von Mises stresses [50]. However, the use of this criterion requires a material with equal compressive and tensile strength, which is in line with cobalt-chromium alloys but differs now from ceramic, tooth tissues and resin composite [51]. To address these issues, a modified von Mises criterion proposed by Williams [52] began to be used, which took into account the ratio of compressive to tensile strengths that was gradually accepted by scholars [44, 53]. In addition, other authors have applied the Tsai-Wu criterion [54], as well as the Christensen criterion [49] for the analysis of brittle materials. Therefore, the judgment of FEA results should be dialectical. Second, this experimental study cannot fully simulate the oral conditions (e.g., oral temperature, humidity, stress fatigue). Besides, the present model involves a simplification of the ideal conditions. There are certain parameters, such as Young’s modulus and Poisson’s ratio of cement materials, that could not be made to correspond to a specific brand of cement in clinical use. Although the results of this study confirm the biomechanical superiority of CGF when restoring residual roots, future research is necessary, especially considering that dentistry is a field involving different clinical situations requiring different methods, and that variations in root canal morphology, amount of tooth tissue remaining, and occlusal forces could all impact on the choice of post-core material. These variables need to be further explored in future experiments. Further experimental research is also needed to identify whether CGF can solve biomechanical problems.

## Conclusion

The FEA results show that CGF can take more stress as Co-Cr in oversized root canals and dumbbell-shaped root canals, resulting in a more uniform stress distribution in the dentin of residual roots. The maximum equivalent von Mises stress and displacement at the adhesive interface were significantly reduced for CGF compared to PGF, suggesting that it may have an advantage for reducing post displacement. Therefore, this material may be one of the best post-core materials for restoring such root canals.

## Data Availability

All data generated or analyzed during this study are included in this published article.
